# Correction: Stereoscopic Analysis of Optic Nerve Head Parameters in Primary Open Angle Glaucoma: The Glaucoma Stereo Analysis Study

**DOI:** 10.1371/journal.pone.0124703

**Published:** 2015-04-07

**Authors:** The PLOS ONE Staff

## Notice of Republication

This article was republished on December 22, 2014 to correct errors in the body of the paper and in Tables 2 and 3 that were introduced during the typesetting process. The publisher apologizes for these errors. Please download this article again to view the correct version. The originally published, uncorrected article and the republished, corrected article are provided here for reference.

## Supporting Information

S1 File Originally published, uncorrected article. (PDF)

S2 File Republished corrected article. (PDF)
