# Supplementary figures and images for: Stereoscopic Analysis of Optic Nerve Head Parameters in Primary Open Angle Glaucoma: The Glaucoma Stereo Analysis Study
Source: PLoS One. 2014 Jun 12;9(6):e99138. doi: 10.1371/journal.pone.0099138 (PMC4055679; doi:10.1371/journal.pone.0099138)

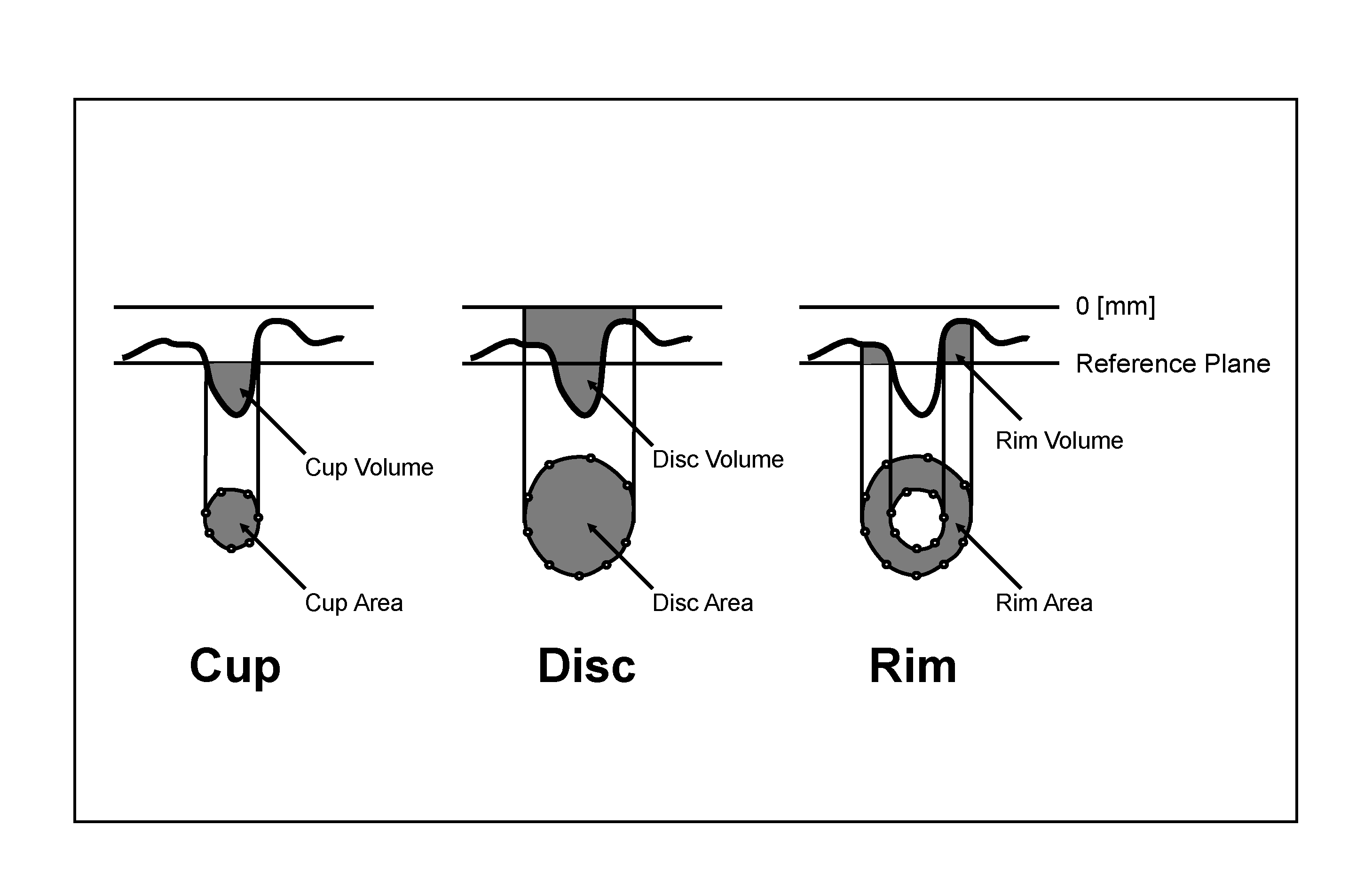

Supplement: Figure S1 — Schemata of measurements for the area and volume of the cup, disc and rim. The reference plane was defined as the average height of the cup contour. The zero-mm plane was defined as the average height of the nasal retinal surface outside the disc area. Contour lines for the disc and cup were based on observer-determined points on the fundus photograph (typically 8–14,) with computer-generated spline interpolation used to generate the final curve. The area and volume of the cup, disc and rim were determined with these two contour lines and the two planes, as illustrated. The superior and the inferior rim widths were measured on the vertical axis of the optic nerve head. In the cup area, the maximum depth and the mean depth were calculated. The height variation contour was calculated by subtracting the minimum height of the disc contour line from its maximum height. Maximum height and minimum height of the depth map were defined as the maximum height and minimum height of the measurement area. (TIF) [file pone.0099138.s001.tif]

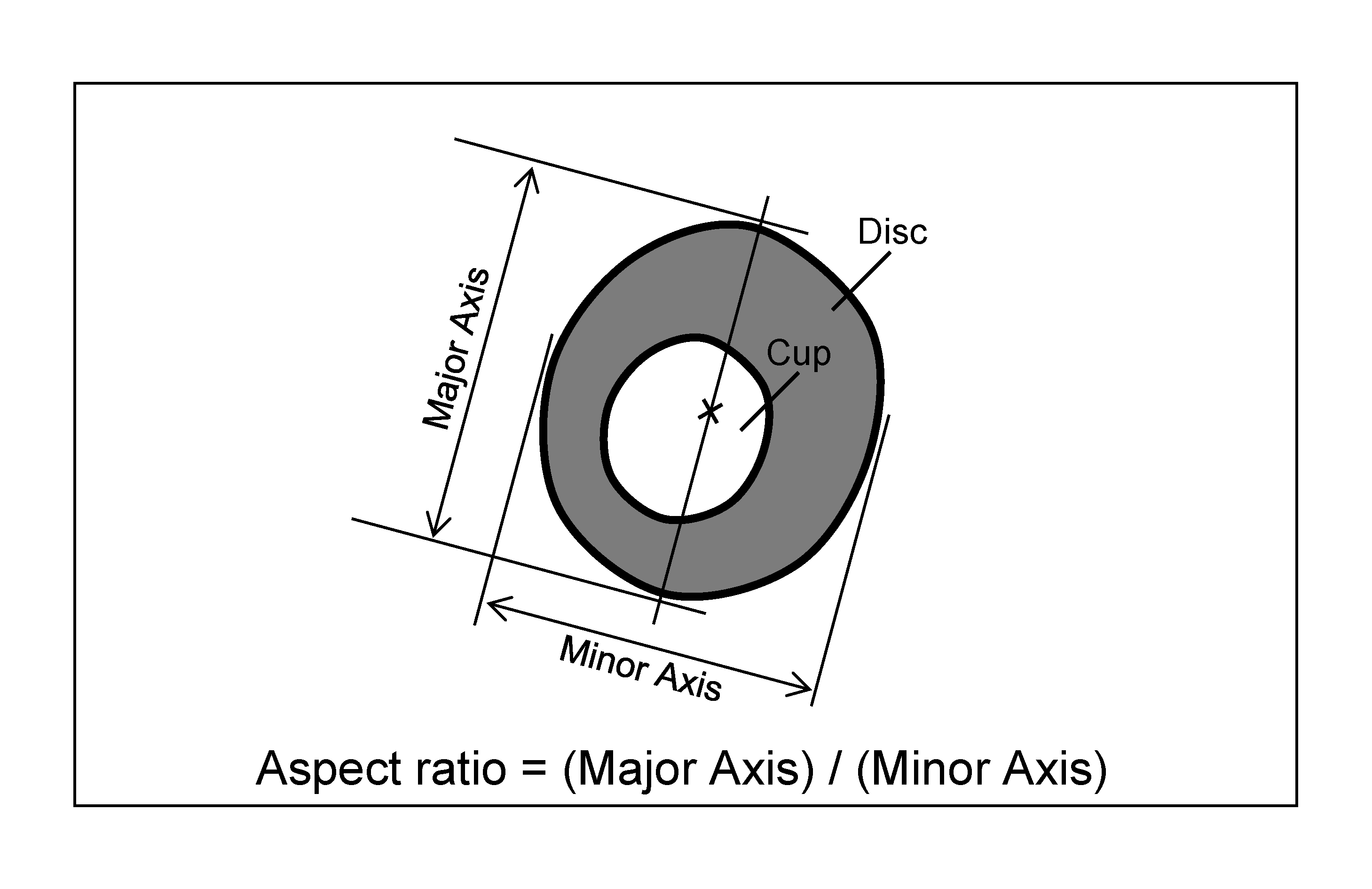

Supplement: Figure S2 — Schema of the aspect ratio. The aspect ratio was calculated by dividing the length of the largest diameter (the major axis) of the optic disc by the perpendicular diameter (the minor axis). (TIF) [file pone.0099138.s002.tif]

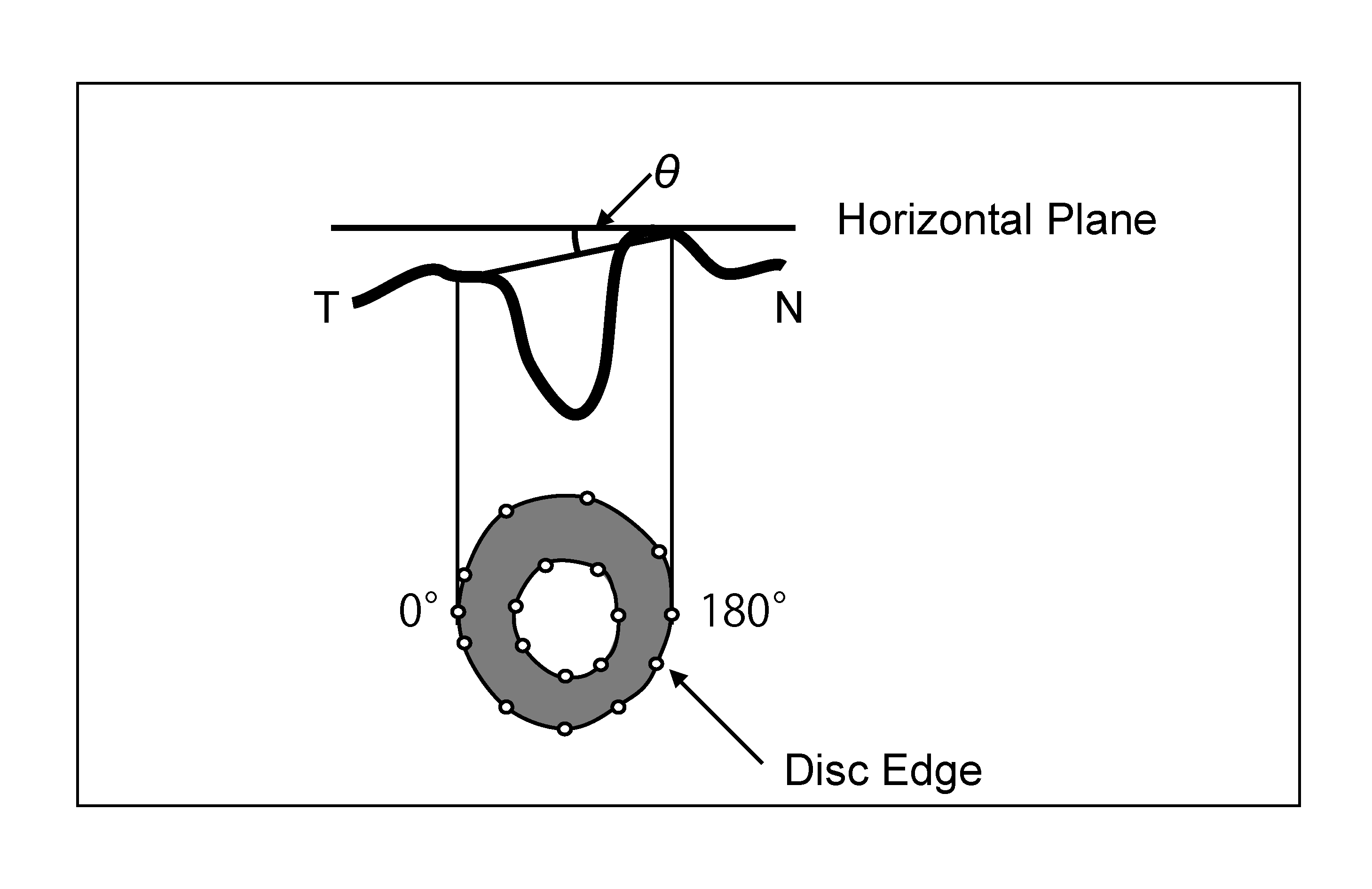

Supplement: Figure S4 — Definition of disc tilt angle. The disc tilt angle was defined as the degree of the angle between the plane horizontal to the observer and the line between 0° and 180° on the disc edge. T: Temporal, N: Nasal, θ: disc tilt angle. (TIF) [file pone.0099138.s004.tif]
