# Supplementary material for: Correction: Stereoscopic Analysis of Optic Nerve Head Parameters in Primary Open Angle Glaucoma: The Glaucoma Stereo Analysis Study
Source: PLoS One. 2015 Apr 7;10(4):e0124703. doi: 10.1371/journal.pone.0124703 (PMC4388853; doi:10.1371/journal.pone.0124703)
Supplement: S2 File — (PDF) [file pone.0124703.s002.pdf]

OPEN ACCESS Freely available online

PLOS ONE

Stereoscopic Analysis of Optic Nerve Head Parameters in Primary Open Angle Glaucoma: The Glaucoma Stereo Analysis Study

Yu Yokoyama[1], Masaki Tanito[2], Koji Nitta[3], Maki Katai[4], Yasushi Kitaoka[5], Kazuko Omodaka[1], Satoru Tsuda[1], Toshiaki Nakagawa[6], Toru Nakazawa[1]*

1 Department of Ophthalmology, Tohoku University Graduate School of Medicine, Sendai, Japan, **2** Department of Ophthalmology, Shimane University Faculty of Medicine, Shimane, Japan, **3** Department of Ophthalmology, Fukui-ken Saiseikai Hospital, Fukui, Japan, **4** Department of Ophthalmology, Sapporo Teishin Hospital, Sapporo, Japan, **5** Department of Ophthalmology, St. Marianna University School of Medicine, Kawasaki, Japan, **6** Kowa Company, Ltd., Nagoya, Japan

Abstract

Purpose: The Glaucoma Stereo Analysis Study (GSAS), a cross sectional multicenter collaborative study, used a stereo fundus camera to assess various morphological parameters of the optic nerve head (ONH) in glaucoma patients and investigated the relationships between these parameters and patient characteristics.

Subjects and Methods: The study included 187 eyes of 187 subjects with primary open angle glaucoma or normal tension glaucoma (male: female = 100: 87, age = 61±9 years). Stereo pairs of ONH photographs were made with a stereo fundus camera (nonmyd WX). ONH morphological parameters were calculated with prototype analysis software. In addition to 35 standard parameters, we defined three novel parameters: disc tilt angle, rim decentering, and the absolute value of rim decentering. The correlation between each parameter and patient characteristics was analyzed with Spearman's rank correlation coefficient.

Results: Patient characteristics included refractive error of −3.38±3.75 diopters, intraocular pressure (IOP) of 13.6±2.6 mmHg, and visual field mean deviation (MD) of −4.71±3.26 dB. Representative ONH parameters included a horizontal disc width of 1.66±0.28 mm, vertical disc width of 1.86±0.23 mm, disc area of 2.42±0.63 mm^2, cup area of 1.45±0.57 mm^2, and cup volume of 0.31±0.22 mm^3. Correlation analysis revealed significant negative associations between vertical cup-to-disc ratio (0.82±0.08) and MD (r = −0.40, *P*<0.01) and between disc tilt angle (10.5±12.5 degrees) and refractive error (r = −0.36, *P*<0.01). Seventy-five percent of the eyes had a positive value for rim decentering (0.30±0.42), indicating that rim thinning manifested more often as an inferior lesion than a superior lesion.

Conclusion: We used stereoscopic analysis to establish a database of ONH parameters, which may facilitate future studies of glaucomatous changes in ONH morphology.

Citation: Yokoyama Y, Tanito M, Nitta K, Katai M, Kitaoka Y, et al. (2014) Stereoscopic Analysis of Optic Nerve Head Parameters in Primary Open Angle Glaucoma: The Glaucoma Stereo Analysis Study. PLoS ONE 9(6): e99138. doi:10.1371/journal.pone.0099138

Editor: Demetrios Vavvas, Massachusetts Eye & Ear Infirmary, Harvard Medical School, United States of America

Received March 17, 2014; **Accepted** April 30, 2014; **Published** June 12, 2014

Copyright: © 2014 Yokoyama et al. This is an open-access article distributed under the terms of the Creative Commons Attribution License, which permits unrestricted use, distribution, and reproduction in any medium, provided the original author and source are credited.

Data Availability: The authors confirm that all data underlying the findings are fully available without restriction. All data are included within the Supporting Information files.

Funding: The authors have no support or funding to report.

Competing Interests: The authors have read the journal's policies and note the following conflicts: MT, MK, YK and T.Nakagawa: Provision of equipment used for research by Kowa Company, Ltd. T.Nakagawa is employed by Kowa Company, Ltd. This does not alter the authors' adherence to all the PLOS ONE policies on sharing data and materials.

* Email: ntoru@oph.med.tohoku.ac.jp

Introduction

Glaucoma is characterized by visual field defects that correspond to damaged areas of the optic nerve head (ONH). It affects over 70 million people worldwide, and is the second most common cause of blindness [1,2]. Currently, the only standard treatment to prevent progression of the most common form of the disease, open angle glaucoma (OAG), is maintenance of low intraocular pressure (IOP) with the use of medication or surgery. Although elevated IOP is generally recognized as a major risk factor for glaucoma [3]. It is well known that multiple factors are related to the development and progression of glaucoma. The progression of glaucomatous optic neuropathy (GON) is irreversible, making early diagnosis and treatment critical. Furthermore, assessment of a patient's ONH morphology is an essential part of the correct diagnosis and evaluation of glaucoma.

Generally, changes in ONH morphology, including thinning of the neuronal rim and enlargement of the ONH excavation, precede the progress of visual field defects. Morphological changes in the ONH are therefore considered important early biomarkers of GON and GON progression [4–6]. When identification of morphological changes is included in mass examinations and screenings, it has a positive effect on the early diagnosis of glaucoma [7,8]. Observation of the ONH remains a key part of

Table 1. Inclusion Criteria.

Inclusion Criteria	
·Subjects…	Were 30–80 years old.
	Had visual acuity better than 0.155 (BCVA, LogMAR).
	Had at least one eye with primary open angle glaucoma.
	Had no optic nerve head anomalies (approximately, 2.4< DM/DD <3).
	Had no other disease affecting the visual field.
	Had no conditions that might cause secondary glaucoma.
	Had undergone automated perimetry with the Humphrey Field Analyzer (SITA standard 30–2 or 24–2) in the six months before the test day.
	Had remaining visual fields of at least MD >−12dB in their most recent tests.
	Had been reliably tested with the Humphrey Field Analyzer (SITA standard 30–2) at least six times for more than three years (fixation loss <33%, false positive and false negative <20%).
	Had visual field defects corresponding to glaucomatous optic disc change.
	Had no history of intraocular surgery other than for glaucoma or cataracts.
	Had no history of surgery for the previous three years.
·If both eyes met the inclusion criteria, the eye with more advanced glaucoma was selected.	

BCVA: best corrected visual acuity
DM/DD: disc-macula distance to disc diameter ratio
MD: mean deviation
doi:10.1371/journal.pone.0099138.t001

follow-up care for glaucoma. Occurrences of rim notch, thinning of the local rim and enlargement of the ONH cup are especially important signs of risk [9]. In a previous study of glaucomatous ONH types using Nicolela's classification method, we found that the progression speed of glaucomatous visual field defects differed with ONH types [10]. Identifying certain patterns of glaucomatous ONH morphology may improve diagnostic accuracy and aid in identifying the effects of structural changes on visual function [11,12]. However, when the ONH is assessed with ophthalmoscopy or a subjective examination, ONH cupping and the severity of glaucoma may be underestimated because of the difficulty of stereoscopic viewing [13].

Topographic analysis with a simultaneous stereo fundus camera (nonmyd WX, Kowa Company, Ltd., Japan) is a noninvasive, noncontact imaging technique that does not require pupillary mydriasis. Its reliability has been demonstrated in a previous paper [14]. It is therefore a promising tool for the assessment of ONH morphology. The Glaucoma Stereo Analysis Study (GSAS) is a multicenter study using this technique to assess various morphological parameters of the ONH in Japanese glaucoma patients. In this, the first report from GSAS, we established a database of various ONH parameters.

Subjects and Methods

This study (The Glaucoma Stereo Analysis Study: GSAS) was a cross sectional, multicenter collaborative study. It was approved by the Institutional Review Boards of the Tohoku University Graduate School of Medicine, Shimane University Faculty of Medicine, Fukui-ken Saiseikai Hospital, Sapporo Teishin Hospital, and St. Marianna University School of Medicine. All experimental procedures were conducted in accordance with the tenets set forth in the Declaration of Helsinki. All data collected from the institutions was analyzed anonymously.

One hundred and eighty-seven eyes of 187 patients with normal tension glaucoma (NTG) or primary open angle glaucoma (POAG) were recruited into this study from five institutions: Tohoku University Hospital, the Hospital of Shimane University Faculty of Medicine, Fukui-ken Saiseikai Hospital, Sapporo Teishin Hospital and the Hospital of St. Marianna University School of Medicine. The patients, whose ages ranged from 30 to 80 years, underwent full clinical ophthalmologic evaluations, including testing for visual acuity, refractive error, and intraocular pressure (IOP) with Goldmann applanation tonometry, as well as slit lamp and fundus examinations. At least one measurement of pre-treatment IOP (baseline IOP) was obtained retrospectively. Pre-surgical data on refractive error was also collected from eyes that had undergone refractive procedures such as cataract surgery. Visual field examinations with the Humphrey visual field analyzer (HFA; Carl Zeiss Meditec Inc., Dublin, California) were performed on all subjects within 6 months of recruitment (SITA standard, 30–2 or 24–2). Data from at least six HFA examinations performed over at least the previous three years was also collected retrospectively for each patient. Only reliable visual field data were used, i.e., from examinations with less than 20% false positives, less than 20% false negatives and less than 33% fixation losses. The mean deviation (MD) slope was calculated from these data.

Glaucoma diagnosis was based on the finding of glaucomatous visual field defects in reliable data from an HFA examination, with corresponding GON. GON was defined as an enlarged vertical cup-to-disc (C/D) ratio, narrow neuroretinal rim (rim) width, notching, and nerve fiber layer defects.

Additional inclusion criteria included: 1) best corrected visual acuity of 0.155 or better (LogMAR), 2) no congenital ONH anomalies, 3) ONH size within the typical normal range, defined as a disc-macula distance to disc diameter (DM/DD) ratio between approximately 2.4 and 3.0, 4) no clinically apparent secondary cause of glaucoma and no other disease affecting the visual field, 5) no history of intraocular surgery other than cataract or glaucoma surgery, 6) no history of cataract or glaucoma surgery in the previous three years, and 7) glaucomatous visual field loss more than −12 dB MD. If both eyes met the inclusion criteria, the eye with more advanced glaucoma was selected.

A summary of the inclusion criteria is shown in Table 1.

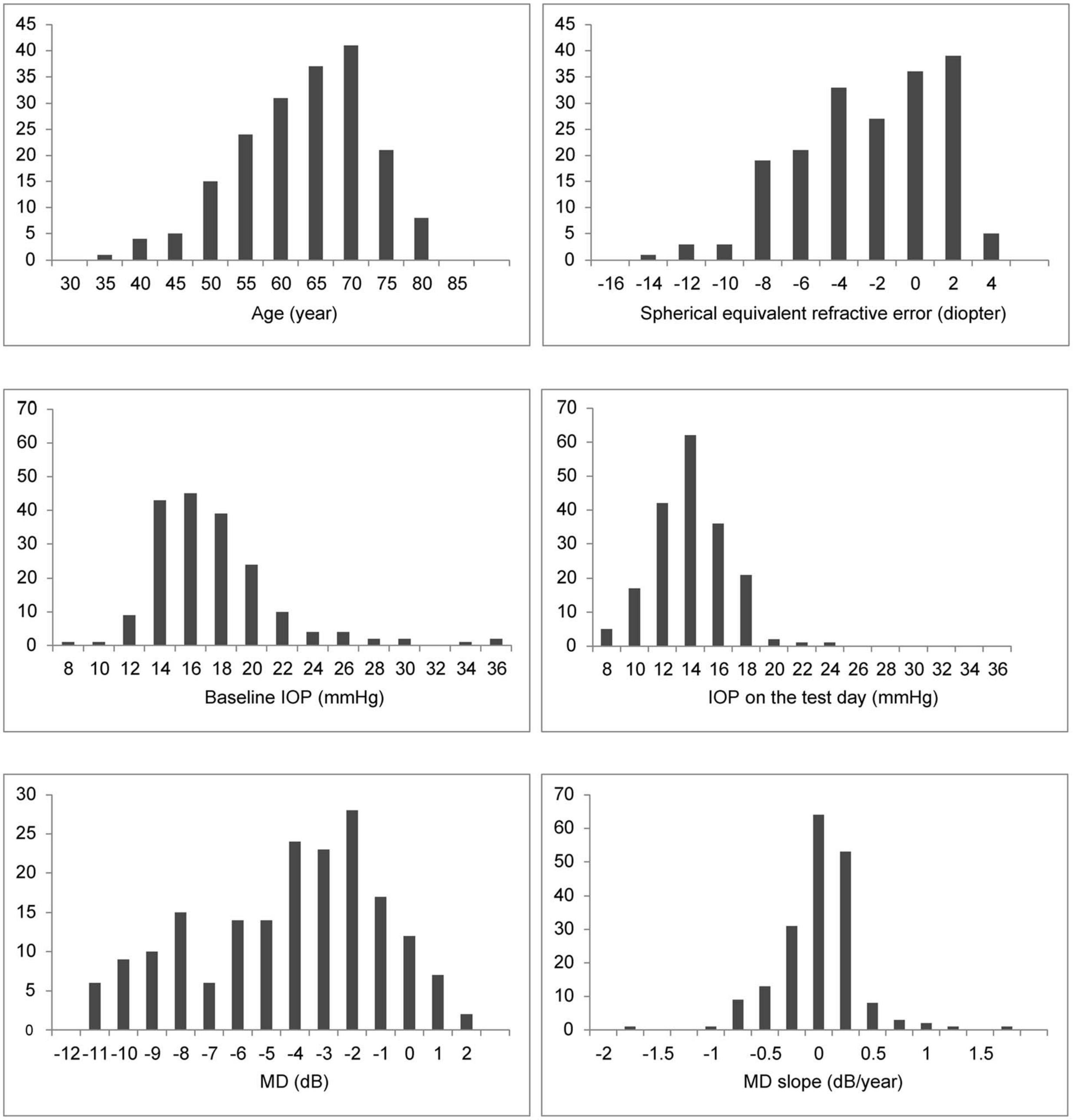

Figure 1. Histograms of ocular characteristics of the subjects. IOP: intraocular pressure, MD: mean deviation
doi:10.1371/journal.pone.0099138.g001

Analysis of optic nerve head topography

Stereo fundus images of the ONH were obtained with a commercially available simultaneous stereo fundus camera (nonmyd WX). The nonmyd WX produces nonmydriatic fundus stereographs, as well as simultaneous right and left parallactic images, by using a single optical system to handle light paths in two directions [14]. The built-in software (VK-2 WX, prototype version, Kowa Company, Ltd., Japan) automatically calculates ONH morphological parameters based on manually-set contour lines for the ONH disc and cup, which in this study were determined by one of the authors (M.T.) while viewing the images stereoscopically. This determination was made according to the recommendations of the Japan Glaucoma Society Guidelines for Glaucoma, 3rd Edition [15,16]. The contour of the disc was delineated by the inner margin of Elschnig's scleral ring, and the contour of the cup was delineated by the outer margin of the cup, which was indicated by the bending of the ONH vessels at the rim. The observer determined several points on the contour (typically 8–14), and the contour line was then automatically generated by software spline interpolation. Parameters nonmyd WX included vertical C/D ratio, upper rim width, lower rim width, cup area, disc area, rim area, C/D area ratio, rim-to-disc (R/D) area ratio, sectional R/D ratio, cup volume, disc volume, rim volume, mean cup depth, maximum cup depth, height variation contour and disc damage likelihood scale (DDLS) stage. Measurements for the area and volume of the cup, disc and rim are illustrated in Figure S1.

Table 2. Subjects demographics.

Characteristics of subjects	
Number	187
Age (year)	61.4 ± 9.4
Sex (male: female)	100 : 87
BCVA (LogMAR)	−0.07 ± 0.08
Refractive error, SE (D)	−3.38 ± 3.75
Baseline IOP (mmHg)	16.9 ± 4.3
IOP on the test day (mmHg)	13.6 ± 2.6
MD (dB)	−4.71 ± 3.26
PSD (dB)	8.08 ± 4.18
MD slope (dB/Y)	−0.12 ± 0.38

BCVA: best corrected visual acuity
SE: spherical equivalent, IOP: intraocular pressure
MD: mean deviation, PSD: pattern standard deviation
doi:10.1371/journal.pone.0099138.t002

Depth value maps, necessary to determine parameters such as disc volume and mean cup depth, were generated based on the disparity between the right and left images of the stereo image pair with a stereo matching technique. Area and volume were calculated using three-dimensional analysis software (VK-2 WX) with correction for magnification. This correction, based a modification of Littman's method, was performed after entering the refractive error and corneal curvature of each eye into the software. The vertical and horizontal C/D ratios were defined by the ratio of the maximal vertical or horizontal diameter of the cup to maximal vertical or horizontal diameter of the disc. The aspect ratio was calculated by dividing the length of the largest diameter of the disc by the diameter perpendicular (Figure S2). DDLS stage (9 stages; 0a, 0b, 1, 2, 3, 4, 5, 6, 7) is a diagnostic parameter proposed by Bayer et al. that provides an estimation of glaucomatous disc damage [17,18]. Analysis of the sectorial rim of the ONH was performed as shown in Figure S3.

In this study, we defined three novel parameters, disc tilt angle, rim decentering, and the absolute value of rim decentering. Rim decentering was calculated with the following formula: rim decentering = (superotemporal rim area − inferotemporal rim area)/(superotemporal rim area + inferotemporal rim area) (Figure S3). For the statistical analysis, absolute values for rim decentering were also determined. Disc tilt angle was defined as the degree of the angle between the horizontal plane and the line drawn from the temporal to the nasal disc edge, passing through the center of the ONH (Figure S4).

Statistical analysis

Statistical analysis was performed with JMP pro 10.02 (SAS Institute Inc.) for Windows. Continuous variables were expressed as mean values ± standard deviation (SD). The Spearman's rank correlation coefficient was used to determine correlations between the patients' characteristics and the measured ONH parameters. In our analysis, ordinal data were treated as continuous data. The level of significance was set at 0.05 in all statistical tests.

Results

The detailed characteristics of the 187 subjects included in this study are shown in Table 2. The average patient age was 61.4±9.3 years. The sex ratio (male to female) was 100: 87, with no significant difference in age between the sexes (60.5±0.9, 62.4±1.0, $P = 0.16$, *t*-test, respectively). The average spherical equivalent refractive error was −3.38±3.75 diopters (if a subject had undergone cataract surgery, pre-surgical refractive error was used to calculate the average), indicating that myopia was common among the subjects in this study. Compared to the baseline (16.9±4.3 mmHg), IOP was lower on the test day (13.6±2.6 mmHg, $P<0.01$, paired *t*-test). The average MD in these eyes was −4.71±3.26 dB. Retrospective data for the follow-up period (82.3±42.7 months, range 36–188 months) showed that the average MD slope was −0.12±0.38 dB/year, with 12% (23 eyes) of subjects progressing at a rate faster than −0.50 dB/year. Histograms are shown in Figure 1.

Average ONH topographic parameters are shown in Table 3. The average disc horizontal and vertical widths were 1.66±0.28 mm and 1.86±0.23 mm, respectively. The average disc aspect ratio was 1.14±0.18. The average size of the disc cup was large (area: 1.45±0.57 mm^2, volume: 0.31±0.22 mm^3) and the average rim of the ONH was thin (area: 0.97±0.27 mm^2, volume: 0.17±0.10 mm^3) in the GON patients. The average DDLS stage (3.77±0.95) indicated that the rim of the ONH had thinned and had defects. We found that 75% of subjects had a positive value (0.30±0.42) for rim decentering, one of the new parameters in this study, indicating that damage to the ONH occurred at the lower side of the rim. The average disc tilt angle was 10.5±12.5 degrees.

To investigate the relationship between ONH morphological parameters, the characteristics of the patients and visual field defects, we used Spearman's rank correlation coefficient (Table 4). Vertical C/D ratio and rim area were significantly correlated to visual field loss (MD) ($r = -0.40$, $P<0.01$, $r = 0.40$, $P<0.01$). Disc tilt angle was significantly correlated to age and spherical equivalent refractive error ($r = -0.30$, $P<0.01$, $r = -0.36$, $P<0.01$). We also performed a multiple regression analysis, assigning tilt angle as the response variable and each of the latter two parameters as explanatory variables, to determine the strongest correlation between disc tilt angle, age and refractive error. The standardized partial regression coefficient (β value) for age was −0.17 ($P = 0.03$), and the variance inflation factor was 1.15. The β value for refractive error was −0.24 ($P<0.01$). We thus concluded that myopia had a stronger impact on disc tilt angle than age.

Table 3. Optic nerve head parameters.

Parameters	mean ±SD	Parameters	mean ±SD
Vertical disc width (mm)	1.86±0.23	Rim-disc ratio of section 1	0.07±0.05
Horizontal disc width (mm)	1.66±0.28	Rim-disc ratio of section 2	0.11±0.06
Vertical cup-disc ratio	0.82±0.08	Rim-disc ratio of section 3	0.17±0.06
Horizontal cup-disc ratio	0.74±0.08	Rim-disc ratio of section 4	0.20±0.08
Minimum rim-disc ratio	0.02±0.02	Rim-disc ratio of section 5	0.15±0.08
Angle of minimum rim-disc ratio (degree)	187.4±127.0	Rim-disc ratio of section 6	0.06±0.05
Superior minimum rim-disc ratio	0.08±0.06	Cup volume (mm^3)	0.31±0.22
Angle of superior minimum rim-disc ratio (degree)	72.9±18.7	Disc volume (mm^3)	0.93±0.43
Inferior minimum rim-disc ratio	0.04±0.05	Rim volume (mm^3)	0.17±0.10
Angle of inferior minimum rim-disc ratio (degree)	284.1±14.9	Mean cup depth (mm)	0.20±0.09
Disc aspect ratio	1.14±0.18	Maximum cup depth (mm)	0.52±0.19
Cup aspect ratio	1.28±0.24	Height variation contour (mm)	0.58±0.26
Superior rim width (mm)	0.21±0.10	Maximum depth value of the depth map (mm)	0.85±0.28
Inferior rim width (mm)	0.12±0.09	Minimum depth value of the depth map (mm)	−0.14±0.25
Cup area (mm^2)	1.45±0.57	Rim category	5.77±0.95
Disc area (mm^2)	2.42±0.63	DDLS stage	3.77±0.95
Rim area (mm^2)	0.97±0.27	Rim decentering	0.30±0.42
Cup-disc area ratio	0.58±0.11	Disc tilt angle (degree)	10.5±12.5
Rim-disc area ratio	0.42±0.11	Rim decentering (absolute value)	0.44±0.27

SD: standard deviation
doi:10.1371/journal.pone.0099138.t003

Table 4. Correlation between optic nerve head parameters and glaucoma patient data.

Parameters	Age (year)		BCVA (LogMAR)		Spherical equivalent refractive error (D)		Pretreatment IOP (mmHg)		IOP on the test day (mmHg)		MD (dB)		PSD (dB)		MD slope (dB/y)	
	r	*P* value	r	*P* value	r	*P* value	r	*P* value	r	*P* value	r	*P* value	r	*P* value	r	*P* value
Vertical disc width	−0.180	0.014 *	0.025	0.736	−0.137	0.061 *	0.132	0.072	0.165	0.024 *	0.114	0.120	−0.108	0.142	0.212	0.004 **
Horizontal disc width	0.072	0.330	0.087	0.236	0.145	0.047 *	0.081	0.268	0.085	0.249	0.177	0.015 *	−0.229	0.002 **	0.149	0.042 *
Vertical cup-disc ratio	0.130	0.076	0.029	0.689	−0.027	0.713	0.246	0.001 **	0.035	0.633	−0.398	0.000 **	0.329	0.000 **	−0.082	0.265
Horizontal cup-disc ratio	0.103	0.160	0.100	0.173	0.052	0.481	0.199	0.006 **	0.161	0.028 *	−0.167	0.022 *	0.106	0.148	0.040	0.584
Minimum rim-disc ratio	−0.111	0.129	−0.045	0.539	0.082	0.266	−0.157	0.032 *	0.067	0.366	0.236	0.001 **	−0.187	0.011 *	−0.002	0.983
Superior minimum rim-disc ratio	0.072	0.330	0.075	0.310	0.240	0.001 **	−0.099	0.176	0.061	0.403	0.340	0.000 **	−0.343	0.000 **	0.032	0.668
Angle of superior minimum rim-disc ratio	0.062	0.396	−0.004	0.955	0.210	0.004 **	0.039	0.594	0.052	0.479	−0.040	0.583	0.006	0.930	−0.056	0.443
Inferior minimum rim-disc ratio	−0.154	0.035 *	−0.046	0.530	−0.007	0.929	−0.154	0.035 *	0.041	0.573	0.257	0.000 **	−0.205	0.005 **	0.060	0.412
Angle of inferior minimum rim-disc ratio	−0.133	0.070	0.006	0.939	−0.253	0.000 **	−0.043	0.557	−0.020	0.783	0.095	0.196	−0.061	0.410	0.046	0.531
Disc aspect ratio	−0.310	0.000 **	−0.154	0.036 *	−0.330	0.000 **	0.014	0.852	0.010	0.890	−0.146	0.047 *	0.198	0.007 **	−0.001	0.987
Cup aspect ratio	−0.242	0.001 **	−0.162	0.027 *	−0.313	0.000 **	−0.003	0.969	−0.109	0.138	−0.236	0.001 **	0.265	0.000 **	−0.069	0.347
Superior rim width	−0.029	0.695	0.075	0.311	0.104	0.158	−0.115	0.117	0.074	0.316	0.364	0.000 **	−0.347	0.000 **	0.086	0.240
Inferior rim width	−0.285	0.000 **	−0.107	0.144	−0.199	0.006 **	−0.146	0.047 *	−0.002	0.981	0.248	0.001 **	−0.185	0.011 *	0.117	0.112
Cup area	0.089	0.226	0.114	0.120	0.080	0.276	0.188	0.010 **	0.166	0.023 *	−0.011	0.880	−0.049	0.504	0.148	0.043 *
Disc area	−0.003	0.967	0.077	0.294	0.075	0.310	0.115	0.117	0.140	0.057	0.171	0.019 *	−0.200	0.006 **	0.188	0.010 **
Rim area	−0.134	0.068	0.004	0.962	0.030	0.686	−0.129	0.080	−0.032	0.669	0.404	0.000 **	−0.342	0.000 **	0.127	0.084
Cup-disc area ratio	0.159	0.030 *	0.114	0.121	0.070	0.340	0.248	0.001 **	0.138	0.060	−0.262	0.000 **	0.169	0.021 *	0.044	0.553
Rim-disc area ratio	−0.160	0.029 *	−0.114	0.122	−0.073	0.324	−0.246	0.001 **	−0.137	0.062	0.262	0.000 **	−0.167	0.022 *	−0.043	0.562
Rim-disc ratio of section 1	0.097	0.186	−0.073	0.323	0.379	0.000 **	−0.232	0.001 **	−0.078	0.287	0.246	0.001 **	−0.221	0.002 **	−0.095	0.198
Rim-disc ratio of section 2	0.002	0.980	0.023	0.758	0.185	0.011 *	−0.150	0.040 *	0.010	0.889	0.327	0.000 **	−0.342	0.000 **	−0.001	0.990
Rim-disc ratio of section 3	−0.235	0.001 **	−0.074	0.317	−0.198	0.007 **	−0.158	0.031 *	−0.134	0.068	0.120	0.103	−0.039	0.599	−0.087	0.238
Rim-disc ratio of section 4	−0.177	0.016 *	−0.074	0.317	−0.314	0.000 **	−0.091	0.216	−0.177	0.015 *	0.005	0.949	0.081	0.269	−0.007	0.925
Rim-disc ratio of section 5	−0.230	0.002 **	−0.109	0.139	−0.299	0.000 **	−0.115	0.117	−0.115	0.118	0.123	0.094	−0.046	0.531	0.047	0.523
Rim-disc ratio of section 6	−0.164	0.025 *	−0.151	0.039 *	−0.064	0.385	−0.175	0.016 *	−0.018	0.804	0.273	0.000 **	−0.221	0.002 **	0.044	0.554
Cup volume	0.087	0.237	0.079	0.284	0.232	0.001 **	0.123	0.093	0.177	0.015 *	0.057	0.435	−0.143	0.051	0.129	0.079
Disc volume	−0.176	0.016 *	−0.167	0.022 *	0.027	0.715	0.186	0.011 *	0.073	0.321	0.049	0.507	−0.045	0.543	0.119	0.105
Rim volume	−0.338	0.000 **	−0.137	0.062	−0.257	0.000 **	−0.046	0.534	−0.048	0.512	0.084	0.250	−0.034	0.646	0.072	0.327
Mean cup depth	0.085	0.246	0.052	0.479	0.281	0.000 **	0.072	0.328	0.164	0.025 *	0.080	0.279	−0.173	0.018 *	0.089	0.228
Maximum cup depth	0.067	0.361	0.044	0.547	0.237	0.001 **	0.056	0.444	0.130	0.076	0.076	0.301	−0.157	0.032 *	0.055	0.457
Height variation contour	−0.215	0.003 **	0.015	0.834	−0.433	0.000 **	0.091	0.215	0.041	0.579	−0.058	0.433	0.009	0.901	0.036	0.629
Depthmap maximum	−0.141	0.055	−0.156	0.033 *	−0.001	0.987	0.107	0.145	0.055	0.458	0.025	0.731	−0.046	0.534	0.026	0.725
Depthmap minimum	−0.141	0.055	−0.156	0.033 *	−0.001	0.987	0.107	0.145	0.055	0.458	0.025	0.731	−0.046	0.534	0.026	0.725

Table 4. Cont.

Parameters	Age (year)		BCVA (LogMAR)		Spherical equivalent refractive error (D)		Pretreatment IOP (mmHg)		IOP on the test day (mmHg)		MD (dB)		PSD (dB)		MD slope (dB/y)	
	r	*P* value	r	*P* value	r	*P* value	r	*P* value	r	*P* value	r	*P* value	r	*P* value	r	*P* value
Rim category	0.048	0.514	0.049	0.509	−0.121	0.100	0.190	0.009 **	−0.024	0.741	−0.262	0.000 **	0.213	0.003 **	−0.011	0.880
DDLS stage	0.048	0.514	0.049	0.509	−0.121	0.100	0.190	0.009 **	−0.024	0.741	−0.262	0.000 **	0.213	0.003 **	−0.011	0.880
Rim decentering	0.118	0.106	0.123	0.094	0.160	0.029 *	0.088	0.232	0.059	0.419	−0.017	0.822	−0.018	0.805	−0.027	0.717
Disc tilt angle	−0.300	0.000 **	−0.183	0.012 *	−0.361	0.000 **	0.026	0.719	−0.069	0.351	−0.035	0.630	0.068	0.356	0.005	0.946
Rim decentering (absolute value)	0.140	0.056	0.128	0.081	0.163	0.026 *	0.073	0.318	0.022	0.766	−0.101	0.168	0.142	0.053	−0.080	0.277

Spearman's rank correlation coefficient: *; P value<0.05, **; P value <0.01, r: correlation coefficient
BCVA: best corrected visual acuity, IOP: intraocular pressure, MD: mean deviation, PSD: pattern standard deviat.
doi:10.1371/journal.pone.0099138.t004

Discussion

The results of this study indicated that stereoscopically quantified parameters of ONH morphology (in particular, vertical C/D ratio, rim width and rim area) correlated to both the clinical background of glaucoma patients and to HFA MD quantifications of the degree of visual field damage. The reliability of the ONH measurement technique used in this study has already been amply demonstrated [14,19]. We believe that measurement of ONH morphological parameters with a simultaneous stereo fundus camera therefore holds promise in future hospital-based multi-center studies of disc morphology in patients with glaucoma.

Although previous studies have often used stereoscopic photography to analyze glaucoma, they have shown that photographic analysis of the ONH has a poor level of inter-observer consistency, even when the observers are experts [20,21]. This limitation prompted the development of clinical imaging technologies capable of objectively determining ONH morphology, such as Heidelberg retinal tomography (HRT). Meanwhile, however, the reliability of stereoscopic photography has continued to progress. Fundus cameras have become increasingly automated, and computer assisted digital analysis of the ONH has been introduced. Computer assisted techniques for analysis of ONH have improved intra-observer reproducibility and inter-observer consistency [22,23]. One of the latest devices, the nonmyd WX, can produce stereoscopic images with its built-in software and optional polarized filters. These enable examinations made by different observers to provide highly consistent results.

Previously, histological studies have determined that the area of the ONH generally ranges from 2.48 to 2.75 mm^2 [24–26]. Additionally, population-based studies using fundus photography have found that the area of the ONH ranges from 2.09 to 2.94 mm^2 [27–31]. We found that in our patients, the mean disc area was 2.45 mm^2, a result that was consistent with earlier reports. Past population-based studies using fundus photography have found that vertical C/D ratio ranges from 0.43 to 0.56 [27,28,30,32]. We found that mean cup area was 1.45 mm^2 and mean vertical C/D ratio was 0.82, results which indicated glaucomatous enlargement of the cup and which were correlated with MD, PSD, disc area and IOP. The result of 0.58 for C/D area ratio with average MD of −4.71 dB, however, was somewhat inconsistent with past reports. Nakatsue et al. used HRT to determine that the C/D area ratio in NTG patients with an average MD of −8.73 dB was 0.54, and that in POAG patients with an average MD of −9.70 dB it was 0.55 [33]. Additionally, Nouri-Mahdavi et al. used HRT to determine that in POAG patients with moderate glaucoma (average MD of −5.1 dB), mean C/D area ratio was 0.52.

This inconsistency may be because of differences in the clinical backgrounds of the subjects and in methods of ONH assessment. Ocular morphological parameters can also be affected by many other factors, such as subjective bias, choice of measurement instrument, definition of parameters, ethnicity and sex [28,34–37]. Moreover, the sample distribution of glaucoma types may have influenced the values of the ONH parameters. In a past study, we demonstrated the correlation between the severity of glaucomatous damage and different glaucomatous ONH types as classified by Nicolela's system [11,38]. Our report found that discs with generalized enlargement, i.e., those with a diffusely enlarged round cup without focal defects on the disc rim, had the highest correlation to MD. By contrast, eyes with the focal ischemic (FI) disc type had no correlation. FI discs have highly localized thinning of the neuronal rim, with other areas of the neuronal rim being normal [11]. Therefore, to assess glaucomatous deformation

of the ONH, multiple approaches to investigate ONH morphology are needed.

To obtain results reflecting the nature of this morphology, we devised two novel measurement parameters. Rim decentering, one of the new parameters, was calculated with a formula that determined the difference in the rim ratio between the superior and inferior areas. The resulting data suggested that this ratio might help classify eyes with the FI disc type and identify regionally damaged areas in glaucomatous eyes. Seventy-five percent of patients in this study had a positive value for rim decentering, suggesting that the inferior rim of the ONH was more vulnerable to glaucomatous change than the superior. Furthermore, the angle of the inferior minimum rim disc ratio was 284 degrees, which was consistent with rim damage being more common in the inferotemporal disc region [39,40].

The other new measurement parameter used in this study was the tilt angle of the disc. We found that the group of glaucoma patients included in our study showed characteristically steeply sloping ONHs, with an average tilt of 10.5 degrees and a tilt of more than 30 degrees in seven percent of patients. Existing studies use varying definitions of disc tilt. Tay et al. used the index of tilt, which was calculated as the quotient of the minimum diameter of the disc divided by its maximum. They defined ONHs as significantly tilted when they had an index of tilt less than or equal to 0.80 [41]. However, other studies have set 0.70 or 0.75 as the criterion for significant tilt [42,43]. These previous methods of determining tilted discs relied on alternative measurement indices, but 3D photographs are capable of providing a direct value for tilt angle and can help in judging the severity of deformity. We found that tilt angle was moderately correlated to refractive error and age, and a multivariate analysis revealed that refractive error was a stronger indicator of a tilted disc than age. In Asia, the prevalence of myopia is significantly higher than in Western countries [44]. Previous studies have reported that myopia and elongated axial length were associated with a higher prevalence of glaucoma [45–49]. Morphologically, an elongated axial length laterally stretches the choroid and retina. As a result, the ONH tends to develop a myopic type tilted disc with crescent peripapillary atrophy. As our data did not include axial length, our analysis of myopic glaucomatous factors used refractive error, which is correlated with axial length. Nevertheless, we believe that quantification of disc tilt with the nonmyd WX may be able to identify a heightened risk of glaucoma in patients, and form a useful future part of clinical analyses of the ONH.

The stereo fundus camera technique used in this study had limits that may have affected our results. In a few patients, structures such as peripapillary atrophy can reduce the color contrast of Elschnig's scleral ring, rendering the image fuzzy and creating difficulties in determining the contour line of the disc edge. Nevertheless, even in such cases, a stereoscopic image provides more information than a monoscopic image, and should lead to more accurate prognoses. This study was also limited by being hospital-based and retrospective, although it included a relatively high number of patients.

In conclusion, we determined baseline ONH data with a simultaneous stereo fundus camera and identified a number of factors, including vertical C/D ratio, rim width and rim area, which were associated with glaucoma severity in the early and middle stages of the disease. Additionally, we found that tilted discs were correlated to spherical equivalent refractive error. We believe that quantitative data on ONH morphology is a powerful tool for clinical research into glaucoma.

Supporting Information

Figure S1 Schemata of measurements for the area and volume of the cup, disc and rim. The reference plane was defined as the average height of the cup contour. The zero-mm plane was defined as the average height of the nasal retinal surface outside the disc area. Contour lines for the disc and cup were based on observer-determined points on the fundus photograph (typically 8–14,) with computer-generated spline interpolation used to generate the final curve. The area and volume of the cup, disc and rim were determined with these two contour lines and the two planes, as illustrated. The superior and the inferior rim widths were measured on the vertical axis of the optic nerve head. In the cup area, the maximum depth and the mean depth were calculated. The height variation contour was calculated by subtracting the minimum height of the disc contour line from its maximum height. Maximum height and minimum height of the depth map were defined as the maximum height and minimum height of the measurement area.
(TIF)

Figure S2 Schema of the aspect ratio. The aspect ratio was calculated by dividing the length of the largest diameter (the major axis) of the optic disc by the perpendicular diameter (the minor axis).
(TIF)

Figure S3 Illustration of rim sections and rim decentering. a) Definitions of the superior and inferior sections of the rim area. The superior section for measurements ranged from 60° to 120° and the inferior section ranged from 240° to 300°. These sections were used to calculate the minimum rim-to-disc ratio and its angle. b) Definitions of the six sections of the rim area. The six sections of the rim were defined as follows: section 1 ranged from 0° to 45° and from 315° to 360°, section 2 ranged from 45° to 90°, section 3 ranged from 90° to 135°, section 4 ranged from 135° to 225°, section 5 ranged from 225° to 270°, and section 6 ranged from 270° to 315°. These six sections were used to calculate the average of the sectional minimum rim-to-disc ratios. Rim decentering was determined with the following formula: rim decentering = (superotemporal rim area [2] − inferotemporal rim area [6])/(superotemporal rim area [2] + inferotemporal rim area [6]).
(TIF)

Figure S4 Definition of disc tilt angle. The disc tilt angle was defined as the degree of the angle between the plane horizontal to the observer and the line between 0° and 180° on the disc edge. T: Temporal, N: Nasal, θ: disc tilt angle.
(TIF)

Acknowledgments

The authors thank Dr. Kazuichi Maruyama for valuable comments and thank Mr. Tim Hilts for reviewing the manuscript.

Author Contributions

Conceived and designed the experiments: T.Nakagawa KN MK YK T.Nakazawa. Performed the experiments: YY MT KN MK YK KO ST T.Nakazawa. Analyzed the data: YY MT. Contributed reagents/materials/analysis tools: T.Nakagawa. Wrote the paper: YY MT T.Nakagawa T.Nakazawa.

References

1. Quigley HA (1996) Number of people with glaucoma worldwide. Br J Ophthalmol 80: 389–393.
2. Resnikoff S, Pascolini D, Etya'ale D, Kocur I, Pararajasegaram R, et al. (2004) Global data on visual impairment in the year 2002. Bull World Health Organ 82: 844–851.
3. Kass MA, Heuer DK, Higginbotham EJ, Johnson CA, Keltner JL, et al. (2002) The Ocular Hypertension Treatment Study: a randomized trial determines that topical ocular hypotensive medication delays or prevents the onset of primary open-angle glaucoma. Arch Ophthalmol 120: 701–713; discussion 829–730.
4. Martus P, Stroux A, Budde WM, Mardin CY, Korth M, et al. (2005) Predictive factors for progressive optic nerve damage in various types of chronic open-angle glaucoma. Am J Ophthalmol 139: 999–1009.
5. Tezel G, Siegmund KD, Trinkaus K, Wax MB, Kass MA, et al. (2001) Clinical factors associated with progression of glaucomatous optic disc damage in treated patients. Arch Ophthalmol 119: 813–818.
6. Johnson CA, Keltner JL, Krohn MA, Portney GL (1979) Photogrammetry of the optic disc in glaucoma and ocular hypertension with simultaneous stereo photography. Invest Ophthalmol Vis Sci 18: 1252–1263.
7. Robin AL, Quigley HA, Pollack IP, Maumenee AE, Maumenee IH (1979) An analysis of visual acuity, visual fields, and disk cupping in childhood glaucoma. Am J Ophthalmol 88: 847–858.
8. Francis BA, Varma R, Vigen C, Lai MY, Winarko J, et al. (2011) Population and high-risk group screening for glaucoma: the Los Angeles Latino Eye Study. Invest Ophthalmol Vis Sci 52: 6257–6264.
9. Lloyd MJ, Mansberger SL, Fortune BA, Nguyen H, Torres R, et al. (2013) Features of optic disc progression in patients with ocular hypertension and early glaucoma. J Glaucoma 22: 343–348.
10. Nakazawa T, Shimura M, Ryu M, Himori N, Nitta F, et al. (2012) Progression of visual field defects in eyes with different optic disc appearances in patients with normal tension glaucoma. J Glaucoma 21: 426–430.
11. Nicolela MT, Drance SM (1996) Various glaucomatous optic nerve appearances: clinical correlations. Ophthalmology 103: 640–649.
12. Park HY, Lee K, Park CK (2012) Optic disc torsion direction predicts the location of glaucomatous damage in normal-tension glaucoma patients with myopia. Ophthalmology 119: 1844–1851.
13. Wolfs RC, Ramrattan RS, Hofman A, de Jong PT (1999) Cup-to-disc ratio: ophthalmoscopy versus automated measurement in a general population: The Rotterdam Study. Ophthalmology 106: 1597–1601.
14. Asakawa K, Kato S, Shoji N, Morita T, Shimizu K (2013) Evaluation of optic nerve head using a newly developed stereo retinal imaging technique by glaucoma specialist and non-expert-certified orthoptist. J Glaucoma 22: 698–706.
15. (2012) [The Japan Glaucoma Society Guidelines for Glaucoma (3rd Edition)]. Nihon Ganka Gakkai Zasshi 116: 3–46.
16. Gloster J, Parry DG (1974) Use of photographs for measuring cupping in the optic disc. Br J Ophthalmol 58: 850–862.
17. Bayer A, Harasymowycz P, Henderer JD, Steinmann WG, Spaeth GL (2002) Validity of a new disk grading scale for estimating glaucomatous damage: correlation with visual field damage. Am J Ophthalmol 133: 758–763.
18. Henderer JD, Liu C, Kesen M, Altangerel U, Bayer A, et al. (2003) Reliability of the disk damage likelihood scale. Am J Ophthalmol 135: 44–48.
19. Nakagawa T, Suzuki T, Hayashi Y, Mizukusa Y, Hatanaka Y, et al. (2008) Quantitative depth analysis of optic nerve head using stereo retinal fundus image pair. Journal of biomedical optics 13: 064026.
20. Lichter PR (1976) Variability of expert observers in evaluating the optic disc. Trans Am Ophthalmol Soc 74: 532–572.
21. Harper R, Reeves B, Smith G (2000) Observer variability in optic disc assessment: implications for glaucoma shared care. Ophthalmic Physiol Opt 20: 265–273.
22. Jayasundera T, Danesh-Meyer HV, Donaldson M, Gamble G (2005) Agreement between stereoscopic photographs, clinical assessment, Heidelberg retina tomograph and digital stereoscopic optic disc camera in estimating vertical cup:disc ratio. Clin Experiment Ophthalmol 33: 259–263.
23. Tanito M, Sagara T, Takamatsu M, Kiuchi Y, Nakagawa T, et al. (2013) Intraobserver and interobserver agreement of computer software-assisted optic nerve head photoplanimetry. Jpn J Ophthalmol.
24. Quigley HA, Brown AE, Morrison JD, Drance SM (1990) The size and shape of the optic disc in normal human eyes. Arch Ophthalmol 108: 51–57.
25. Jonas JB, Gusek GC, Guggenmoos-Holzmann I, Naumann GO (1988) Size of the optic nerve scleral canal and comparison with intravital determination of optic disc dimensions. Graefe's archive for clinical and experimental ophthalmology = Albrecht von Graefes Archiv fur klinische und experimentelle Ophthalmologie 226: 213–215.
26. Ogden TE, Duggan J, Danley K, Wilcox M, Minckler DS (1988) Morphometry of nerve fiber bundle pores in the optic nerve head of the human. Experimental eye research 46: 559–568.
27. Bourne RR, Foster PJ, Bunce C, Peto T, Hitchings RA, et al. (2008) The morphology of the optic nerve head in the Singaporean Chinese population (the Tanjong Pagar study): part 1—Optic nerve head morphology. Br J Ophthalmol 92: 303–309.
28. Ramrattan RS, Wolfs RC, Jonas JB, Hofman A, de Jong PT (1999) Determinants of optic disc characteristics in a general population: The Rotterdam Study. Ophthalmology 106: 1588–1596.
29. Xu L, Wang Y, Yang H, Zhang L, Jonas JB (2007) Size of the neuroretinal rim and optic cup and their correlations with ocular and general parameters in adult Chinese: the Beijing eye study. Br J Ophthalmol 91: 1616–1619.
30. Varma R, Tielsch JM, Quigley HA, Hilton SC, Katz J, et al. (1994) Race-, age-, gender-, and refractive error-related differences in the normal optic disc. Arch Ophthalmol 112: 1068–1076.
31. Jonas JB, Gusek GC, Naumann GO (1988) Optic disc, cup and neuroretinal rim size, configuration and correlations in normal eyes. Invest Ophthalmol Vis Sci 29: 1151–1158.
32. Crowston JG, Hopley CR, Healey PR, Lee A, Mitchell P (2004) The effect of optic disc diameter on vertical cup to disc ratio percentiles in a population based cohort: the Blue Mountains Eye Study. Br J Ophthalmol 88: 766–770.
33. Nakatsue T, Shirakashi M, Yaoeda K, Funaki S, Funaki H, et al. (2004) Optic disc topography as measured by confocal scanning laser ophthalmoscopy and visual field loss in Japanese patients with primary open-angle or normal-tension glaucoma. J Glaucoma 13: 291–298.
34. Parkin B, Shuttleworth G, Costen M, Davison C (2001) A comparison of stereoscopic and monoscopic evaluation of optic disc topography using a digital optic disc stereo camera. Br J Ophthalmol 85: 1347–1351.
35. Girkin CA, McGwin G, Jr., McNeal SF, DeLeon-Ortega J (2003) Racial differences in the association between optic disc topography and early glaucoma. Invest Ophthalmol Vis Sci 44: 3382–3387.
36. Zangwill LM, Weinreb RN, Berry CC, Smith AR, Dirkes KA, et al. (2004) Racial differences in optic disc topography: baseline results from the confocal scanning laser ophthalmoscopy ancillary study to the ocular hypertension treatment study. Arch Ophthalmol 122: 22–28.
37. Jayasundera T, Danesh-Meyer HV, Donaldson M, Gamble G (2005) Agreement between stereoscopic photographs, clinical assessment, Heidelberg retina tomograph and digital stereoscopic optic disc camera in estimating vertical cup: disc ratio. Clinical and Experimental Ophthalmology 33: 259–263.
38. Omodaka K, Nakazawa T, Otomo T, Nakamura M, Fuse N, et al. (2010) Correlation between morphology of optic disc determined by Heidelberg Retina Tomograph II and visual function in eyes with open-angle glaucoma. Clin Ophthalmol 4: 765–772.
39. Garway-Heath DF, Hitchings RA (1998) Quantitative evaluation of the optic nerve head in early glaucoma. Br J Ophthalmol 82: 352–361.
40. Jonas JB, Fernandez MC, Sturmer J (1993) Pattern of glaucomatous neuroretinal rim loss. Ophthalmology 100: 63–68.
41. Tay E, Seah SK, Chan SP, Lim AT, Chew SJ, et al. (2005) Optic disk ovality as an index of tilt and its relationship to myopia and perimetry. Am J Ophthalmol 139: 247–252.
42. You QS, Xu L, Jonas JB (2008) Tilted optic discs: The Beijing Eye Study. Eye (Lond) 22: 728–729.
43. How AC, Tan GS, Chan YH, Wong TT, Seah SK, et al. (2009) Population prevalence of tilted and torted optic discs among an adult Chinese population in Singapore: the Tanjong Pagar Study. Arch Ophthalmol 127: 894–899.
44. Sawada A, Tomidokoro A, Araie M, Iwase A, Yamamoto T (2008) Refractive errors in an elderly Japanese population: the Tajimi study. Ophthalmology 115: 363–370 e363.
45. Mitchell P, Hourihan F, Sandbach J, Wang JJ (1999) The relationship between glaucoma and myopia: the Blue Mountains Eye Study. Ophthalmology 106: 2010–2015.
46. Grodum K, Heijl A, Bengtsson B (2001) Refractive error and glaucoma. Acta ophthalmologica Scandinavica 79: 560–566.
47. Perera SA, Wong TY, Tay WT, Foster PJ, Saw SM, et al. (2010) Refractive error, axial dimensions, and primary open-angle glaucoma: the Singapore Malay Eye Study. Arch Ophthalmol 128: 900–905.
48. Xu L, Wang Y, Wang S, Jonas JB (2007) High myopia and glaucoma susceptibility the Beijing Eye Study. Ophthalmology 114: 216–220.
49. Suzuki Y, Iwase A, Araie M, Yamamoto T, Abe H, et al. (2006) Risk factors for open-angle glaucoma in a Japanese population: the Tajimi Study. Ophthalmology 113: 1613–1617.
